# Mesial Inclination of Bur for the Sectioning of Impacted Distoangular Mandibular Third Molar: A Novel Technique

**DOI:** 10.7759/cureus.66556

**Published:** 2024-08-10

**Authors:** Nimish Situt, Ramakrishna Shenoi, Alvina V Waghchoure, Kshitij Bang

**Affiliations:** 1 Oral and Maxillofacial Surgery, VSPM's Dental College and Research Center, Nagpur, IND

**Keywords:** atraumatic third molar extraction, impacted mandibular third molar, disimpaction, wisdom tooth surgery, distoangular third molar

## Abstract

Distoangular impacted teeth account for about 4.8% of all impacted tooth angulations. Various indices in the literature used to assess the difficulty of surgically extracting impacted third molars indicate that distoangular impactions are the most complex. This complexity necessitates the development of specific skills by the operator. The difficulty arises primarily due to the challenging position of these teeth, which complicates access and instrumentation. The proposed method aims to simplify the sectioning process for distoangular third molars by avoiding unnecessary buccal bone removal, improving the accuracy of root sectioning, and preserving buccal bone. Consequently, this technique reduces postoperative pain and swelling, resulting in better patient outcomes.

## Introduction

Archer has defined impaction as “the tooth, which fails to erupt in the oral cavity in its functional position and which has lost its further potential of eruption.” The etiology pertaining to the impaction of these third molars is multifactorial which includes lack of space in the maxilla or the mandible for the eruption of these third molars, pathological lesions, dense amount of bone covering the tooth, aberrant path of the eruption, position of the tooth bud not favoring its eruption [1]. One of the etiology is the distal direction of the eruption of these third molars. The incidence of distoangular impacted teeth accounts for about 4.8% of the different angulations of the impacted teeth [2]. According to various indices mentioned in the literature to assess the difficulty of surgical extraction of impacted third molar, distoangular accounts for the highest value in the index, suggesting the complexity it poses for the surgical extraction of the tooth and its requirement for the operator to attain skills for surgical extraction of these teeth [3-5]. One of the reasons that poses a difficulty to the operator for the extraction of these impacted teeth is the position of these teeth which elicits a difficulty in gaining access to these teeth and instrumentation. The use of air-driven handpieces puts the patients in jeopardy of developing air emphysema as the lower molar roots are located anatomically in close proximity to the fascial planes and spaces [2]. This necessitates the use of non-air-driven handpieces for surgical extractions which eventually leads to difficulty in extracting these third molars.

This proposed method seeks to simplify the process of sectioning distoangular third molars. It minimizes unnecessary removal of buccal bone, it increases the precision of root sectioning while preserving buccal bone. As a result, it minimizes postoperative pain and swelling, leading to better patient outcomes.

## Technical report

**Figure 1:**
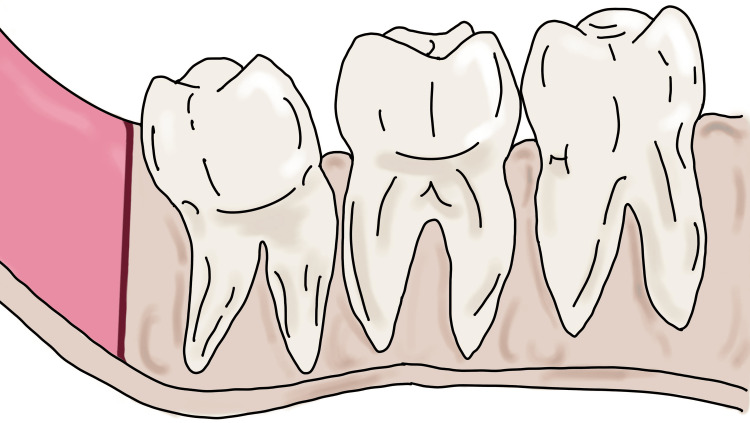
Depiction of distoangular third molar with a path of exit toward ramus and less interdental bone between third and second molar Image Credit: Nimish Situt

We propose a technique for the extraction of the distoangular third molar (Figure 1).

After the administration of local anesthesia, a mucoperiosteal flap is raised according to the convenience of the clinician for gaining adequate access to the tooth that is to be extracted. With the help of a 7/8 round bur and a straight bur no. 702 or 703 is used for the removal of the bone. To gain an orientation to the crown structure, the bur should be used in a sweeping motion to remove the optimal amount of bone from the occlusal, buccal, and distal aspects of the tooth. Following exposure of the crown structure of the tooth, the Moore Gillbe Collar technique is used to create a buccal trough/gutter around the tooth. Care must be taken that the buccal trough is created in the cancellous bone. After creating the trough, sectioning of the tooth is an important aspect of this technique to reduce the amount of bone that is usually required to remove the distoangular third molar.

**Figure 2:**
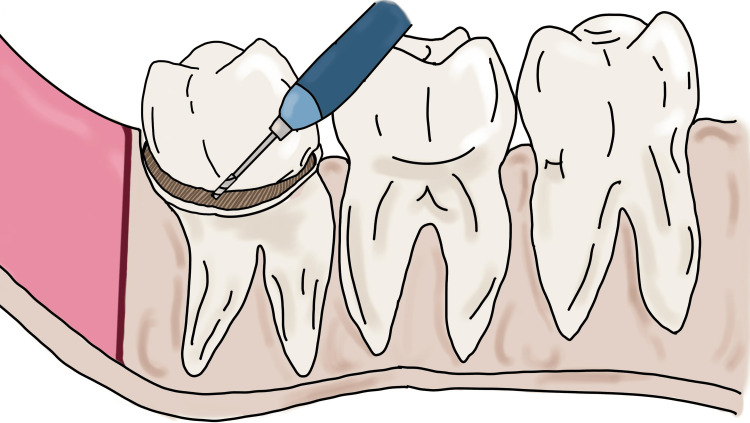
Depiction of the coronectomy procedure where the crown is separated from the radicular portion of the tooth Image Credit: Nimish Situt

Coronectomy is initiated by separating the crown from the radicular portion by horizontal sectioning of the tooth (Figure 2).

**Figure 3:**
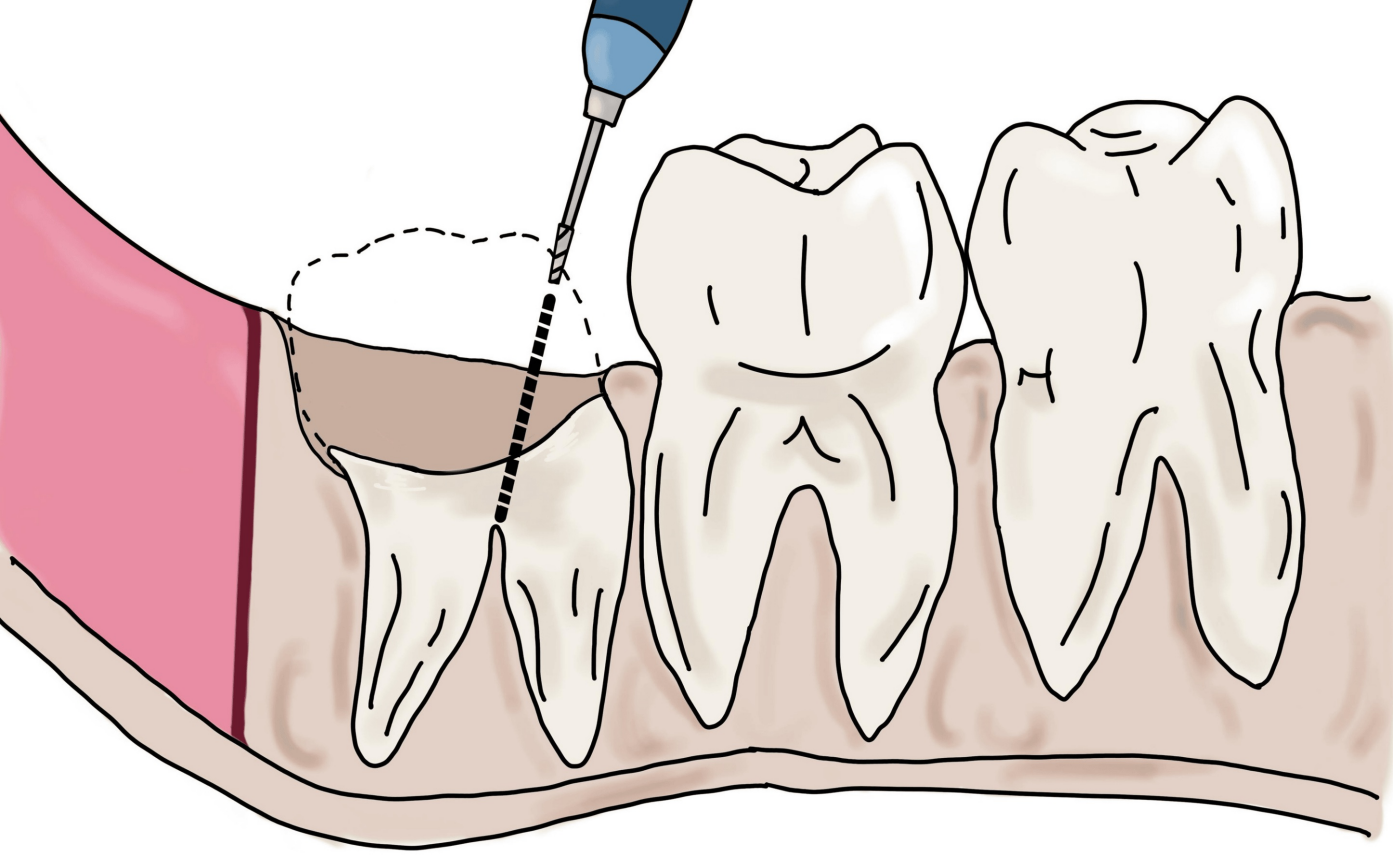
Sectioning of the roots and placement of the bur from the mesial third of the radicular portion Image Credit: Nimish Situt

After horizontal sectioning, the remaining radicular portion of the tooth is visualized into three parts that is the distal third, the middle third, and the mesial third of the tooth. The straight bur no. 702 is placed from the mesial third of the remaining radicular portion and the bur is angulated such that the tip of the bur reaches the furcation area of the roots (Figure 3).

**Figure 4:**
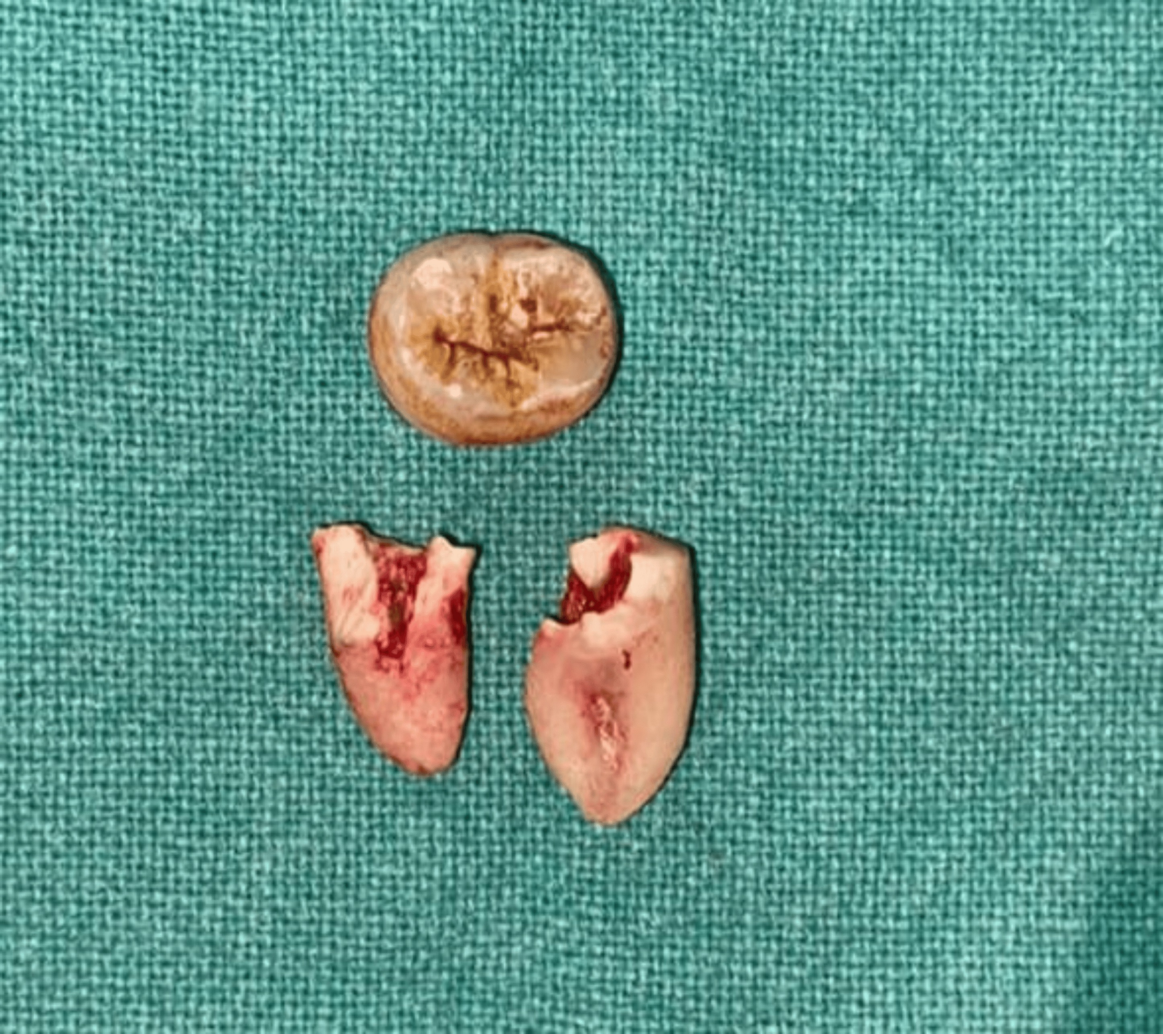
Depiction of sectioning of the crown done by coronectomy procedure and sectioning of the roots exactly at the bifurcation Image Credit: Alvina Waghchoure

This leads to proper sectioning of the roots of the distoangular tooth. After this, the separated root stumps are elevated separately and extracted according to their path of removal (Figure 4).

Care must be taken that the bur is placed from the mesial third of the remaining radicular portion and not from the middle third. If the bur is placed from the middle third portion, this leads to improper sectioning of the roots distal to the intended furcation area. For precise application of this technique, it is important that the motor-driven instrument is angulated at a proper position. As it is difficult to angulate the straight handpiece at 90 degrees from the middle third, the author suggests the instrument be angulated from the mesial third as close as possible to 90 degrees so as to reach the intended furcation area.

## Discussion

Distoangular impacted teeth are difficult to extract because they tend to be delivered posteriorly during extraction due to their arc of rotation and the lack of interdental space for the elevator application [6]. Effective tooth sectioning helps in removing the tooth while minimizing bone removal [7]. Various studies have shown that distoangular impactions of third molars are rated as the most challenging to extract surgically. This highlights the considerable complexity involved in these cases and emphasizes the importance of practitioners acquiring specialized techniques for their extraction [8-10].

The positioning of these impacted teeth complicates access and instrumentation for the operator. Moreover, the use of air-driven handpieces can pose a risk of air emphysema, especially since the lower molar roots are in close proximity to fascial planes and spaces [2]. To mitigate this risk, non-air-driven handpieces are recommended during surgical extractions, further complicating the removal of these third molars. Therefore, this proposed technique aims to simplify the sectioning of distoangular third molars. This technique avoids unnecessary buccal bone removal, enhances the precision of root sectioning, and conserves buccal bone. Consequently, it reduces postoperative pain and swelling, improving patient outcomes.

This technique offers numerous advantages. First, it helps in preserving the distolingual alveolar bone, which is crucial for maintaining the structural integrity of the lingual cortex. Additionally, it significantly reduces the chances of injuring the lingual nerve, making it a safer option compared to other techniques. The method also facilitates the easy removal of root stumps, allowing for different paths of removal, which enhances its versatility. This technique is particularly beneficial for dealing with roots that have varying morphologies, often posing a challenge during extractions. Furthermore, it is suitable for extracting grossly carious teeth and bulbous teeth, which are typically difficult to manage with standard extraction techniques.

However, there are limitations to this method. It is not suitable for patients with limited mouth opening, as an adequate amount of mouth opening is necessary to achieve the proper angulation of the instrument. If the bur is placed from the middle third of the tooth, it can lead to improper sectioning distal to the intended furcation area. Therefore, it is crucial to angulate the bur from the mesial third and maintain the handpiece at an angle close to 90 degrees to ensure accurate sectioning. This requirement for precise angulation can be a limiting factor, that makes access difficult.

## Conclusions

In conclusion, distoangular impacted third molars present significant extraction challenges due to their positioning and difficulty of access. The proposed technique, by enhancing root sectioning accuracy and preserving buccal bone, offers a substantial improvement in surgical outcomes. This method not only simplifies the extraction process but also minimizes postoperative pain and swelling, thereby improving overall patient comfort and recovery. The authors suggest the proposed technique be conducted on a larger cohort to know the efficacy and advantages of this technique in comparison to other techniques used for extraction of the distoangular third molar.

## References

[1] Passi D, Singh G, Dutta S (2019). Study of pattern and prevalence of mandibular impacted third molar among Delhi-National Capital Region population with newer proposed classification of mandibular impacted third molar: a retrospective study. Natl J Maxillofac Surg.

[2] Mascarenhas RJ (2019). Management of subcutaneous facial emphysema secondary to a class V dental restoration. Clin Case Rep.

[3] Kim JY, Yong HS, Park KH, Huh JK (2019). Modified difficult index adding extremely difficult for fully impacted mandibular third molar extraction. J Korean Assoc Oral Maxillofac Surg.

[4] Shaari RB, Awang Nawi MA, Khaleel AK, AlRifai AS (2023). Prevalence and pattern of third molars impaction: a retrospective radiographic study. J Adv Pharm Technol Res.

[5] Al-Madani SO, Jaber M, Prasad P, Maslamani MJ (2024). The patterns of impacted third molars and their associated pathologies: a retrospective observational study of 704 patients. J Clin Med.

[6] Kumar KS, Sargunam AE, Ravindran C, Giri G (2018). Intra-alveolar extraction of impacted distoangular mandibular third molars: a novel technique. Indian J Dent Res.

[7] Agarwal SS, Xavier F, Rao S, Galhotra V (2023). Does the tooth sectioning method impact surgical removal of the distoangular impacted mandibular third molar?. J Oral Maxillofac Surg.

[8] Marciani RD (2007). Third molar removal: an overview of indications, imaging, evaluation, and assessment of risk. Oral Maxillofac Surg Clin North Am.

[9] Bokindo IK, Butt F, Macigo F (2017). Variant root morphology of third mandibular molar in normal and impacted teeth. Anat J Afr.

[10] Farish SE, Bouloux GF (2007). General technique of third molar removal. Oral Maxillofac Surg Clin North Am.

